# Supplementary material for: Consortia of anti-nematode fungi and bacteria in the rhizosphere of soybean plants attacked by root-knot nematodes
Source: R Soc Open Sci. 2019 Mar 27;6(3):181693. doi: 10.1098/rsos.181693 (PMC6458363; doi:10.1098/rsos.181693)
Supplement: Figure S1. [file rsos181693supp1.pdf]

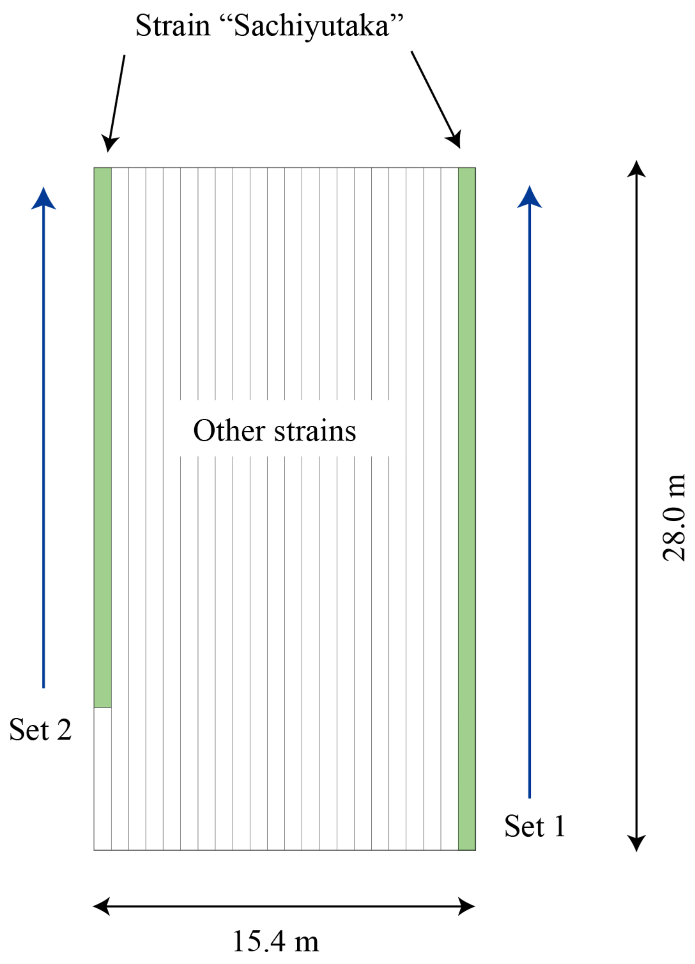

Figure S1. Map of the soybean field. Terminal roots and rhizosphere soil of the soybean strain “Sachiyutaka” were sampled from the two lines indicated in the map.
