# Supplementary figures and images for: Consortia of anti-nematode fungi and bacteria in the rhizosphere of soybean plants attacked by root-knot nematodes
Source: R Soc Open Sci. 2019 Mar 27;6(3):181693. doi: 10.1098/rsos.181693 (PMC6458363; doi:10.1098/rsos.181693)

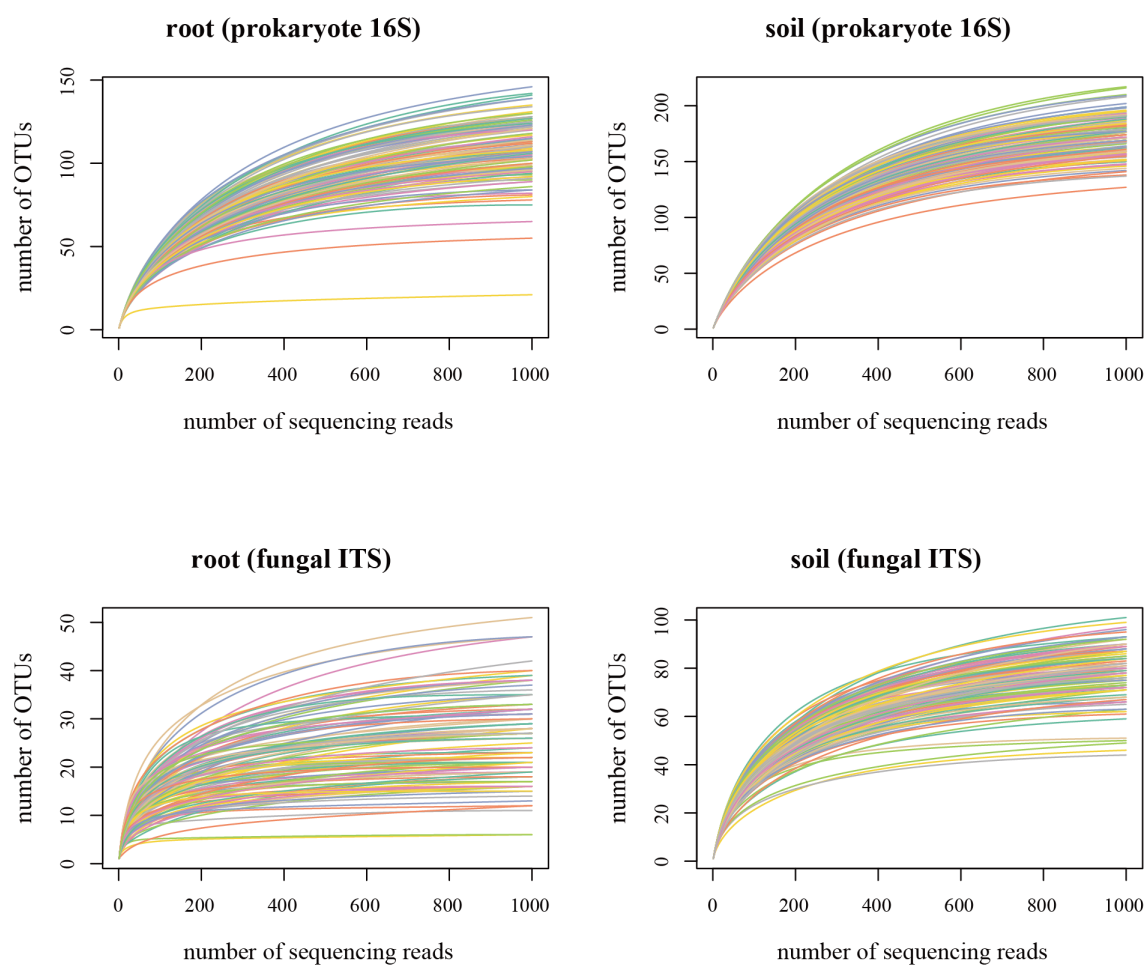

**Figure S2.** Relationship between the number of sequencing reads and that of OTUs.

Supplement: Figure S2. [file rsos181693supp2.pdf]
