# Supplementary material for: Consortia of anti-nematode fungi and bacteria in the rhizosphere of soybean plants attacked by root-knot nematodes
Source: R Soc Open Sci. 2019 Mar 27;6(3):181693. doi: 10.1098/rsos.181693 (PMC6458363; doi:10.1098/rsos.181693)
Supplement: Figure S3. [file rsos181693supp3.pdf]

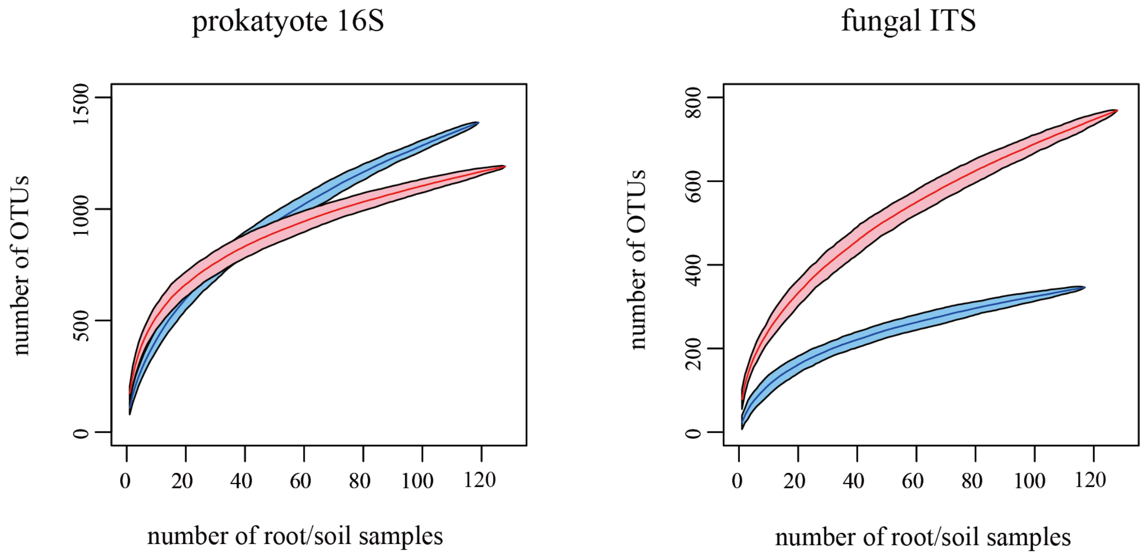

Figure S3. Relationship between the number of root/soil samples and that of prokaryote/fungal OTUs. Blue and red curves represent root and soil samples, respectively.
