# Supplementary material for: Consortia of anti-nematode fungi and bacteria in the rhizosphere of soybean plants attacked by root-knot nematodes
Source: R Soc Open Sci. 2019 Mar 27;6(3):181693. doi: 10.1098/rsos.181693 (PMC6458363; doi:10.1098/rsos.181693)
Supplement: Figure S4. [file rsos181693supp4.pdf]

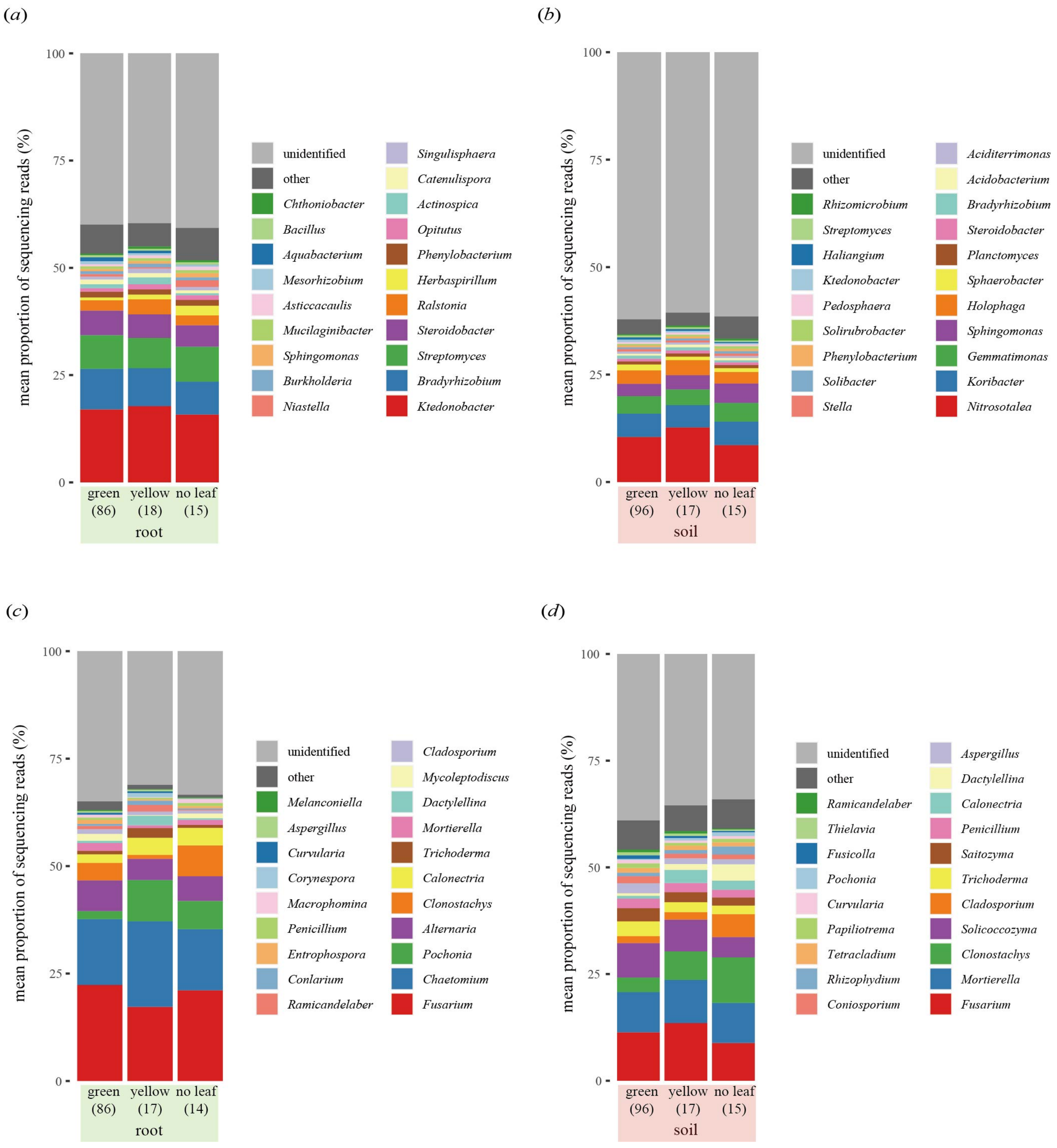

Figure S4. Genus-level compositions of prokaryote and fungal community structure. (*a*) Prokaryotes in the root dataset. Mean proportions of sequencing reads are shown for respective taxa. The numbers of the samples from which sequencing data were successfully obtained are shown in the parentheses. (*b*) Prokaryotes in the soil dataset. (*c*) Fungi in the root dataset. (*d*) Fungi in the soil dataset.
