# Supplementary material for: Consortia of anti-nematode fungi and bacteria in the rhizosphere of soybean plants attacked by root-knot nematodes
Source: R Soc Open Sci. 2019 Mar 27;6(3):181693. doi: 10.1098/rsos.181693 (PMC6458363; doi:10.1098/rsos.181693)
Supplement: Figure S5. [file rsos181693supp5.pdf]

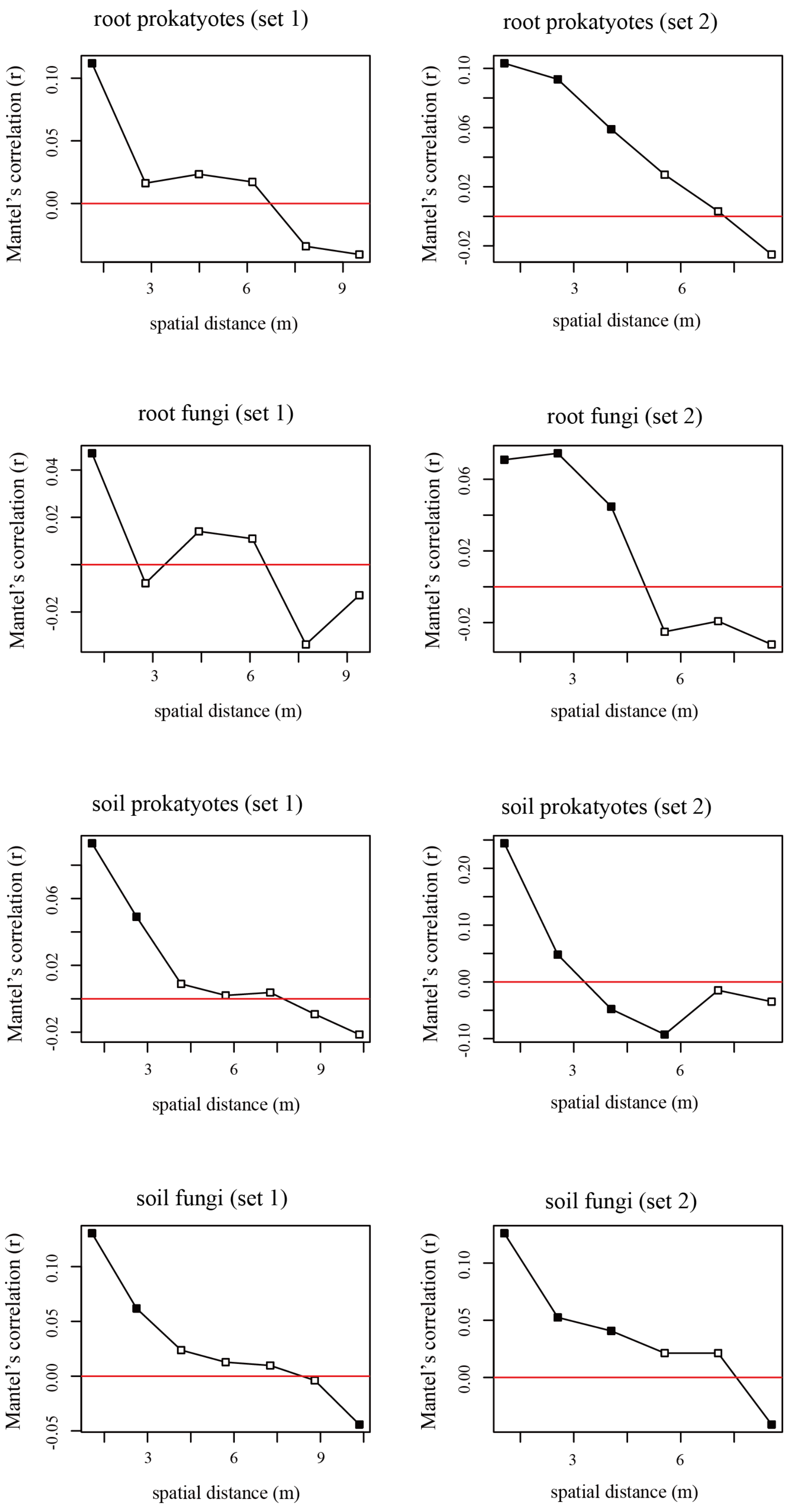
root prokatyotes (set 1)
root prokatyotes (set 2)
root fungi (set 1)
root fungi (set 2)
soil prokatyotes (set 1)
soil prokatyotes (set 2)
soil fungi (set 1)
soil fungi (set 2)
Mantel's correlation (r)
spatial distance (m)
-0.10
-0.05
-0.02
0.00
0.02
0.04
0.05
0.06
0.10
0.20
3
6
9

Figure S5. Spatial autocorrelation of prokaryote/fungal community structure. A positive value indicated by filled squares represents statistically significant spatial autocorrelation at the spatial distance class ($\alpha = 0.05$). Results for sampling sets 1 and 2 are shown separately.
