# Supplementary material for: Consortia of anti-nematode fungi and bacteria in the rhizosphere of soybean plants attacked by root-knot nematodes
Source: R Soc Open Sci. 2019 Mar 27;6(3):181693. doi: 10.1098/rsos.181693 (PMC6458363; doi:10.1098/rsos.181693)
Supplement: Figure S6. [file rsos181693supp6.pdf]

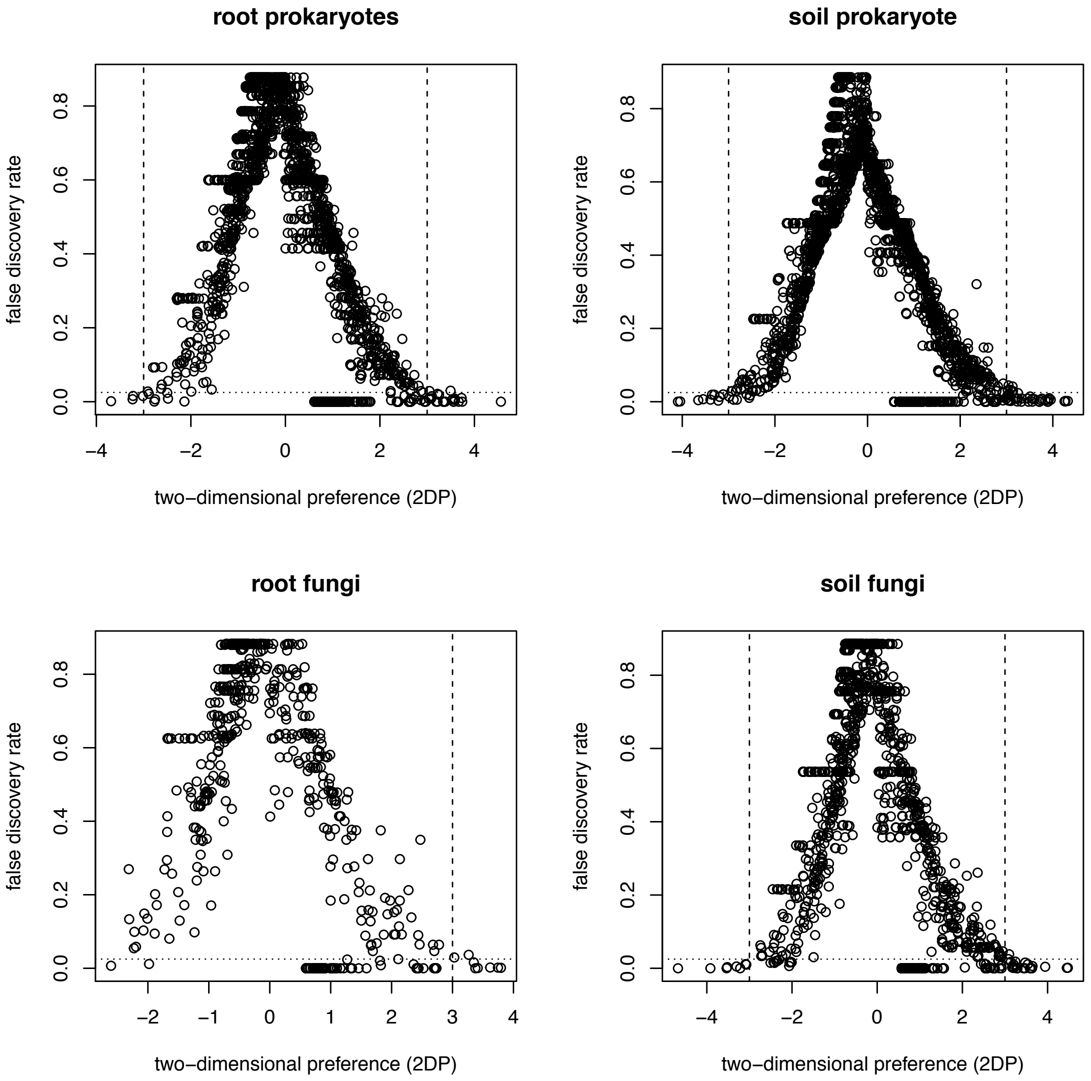

Figure S6. Relationship between preference index values and false discovery rates in the randomization analysis for screening host-state-specific OTUs.
